# Facile Synthesis of Vertically Arranged CNTs for Efficient Solar-Driven Interfacial Water Evaporation

**DOI:** 10.1021/acsomega.2c06706

**Published:** 2022-12-12

**Authors:** Lifen Su, Xiaoyu Liu, Xu Li, Bin Yang, Bin Wu, Ru Xia, Jiasheng Qian, Jianhua Zhou, Lei Miao

**Affiliations:** † Anhui Province Key Laboratory of Environment-Friendly Polymer Materials, School of Chemistry and Chemical Engineering, Anhui University, Hefei230601, China; ‡ School of Materials Science and Engineering, Anhui University, Hefei230601, China; § Guangxi Key Laboratory of Information Materials, Engineering Research Center of Electronic Information Materials and Devices, Ministry of Education, Guilin University of Electronic Technology, Guilin541004, China

## Abstract

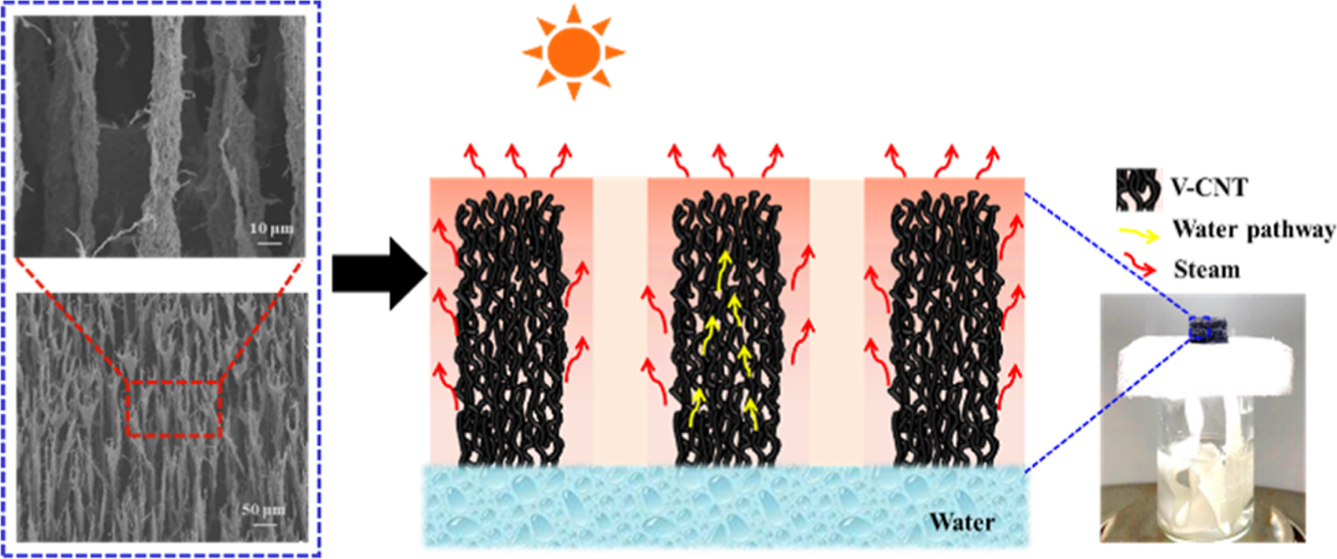

Solar-driven evaporation of water is a sustainable and promising technology for addressing the crisis of clean water. Herein, novel vertically arranged carbon nanotube (V-CNT) aerogels with a tree branch structure is facilely synthesized through an ice templating method. The V-CNT-based photothermal evaporator exhibits efficient broadband light trapping and super-hydrophilicity. Owing to the unique structure and ultrafast water transportation, a high evaporation rate of 3.26 kg m^–2^ h^–1^ was achieved by the three-dimensional V-CNT-based evaporator under a solar illumination of 1 kW m^–2^. More significantly, the V-CNT shows excellent recycling stability and salt-resistant performance in seawater and may provide a novel strategy to the practical sustainable technique of water purification applications.

## 1 Introduction

Freshwater and energy are the most important strategic resources for the development of economies and countries in the word.^1−3^ With the rapid development of society, shortage of freshwater resource is a challenge for humans. Using the abundant and free solar energy to produce clean water is a perfect solution to address the freshwater crisis.^4,5^ The traditional solar-driven purification technology calls for expensive device for collecting solar energy to heat the bulk water and frequent maintenance, resulting in high cost and low photothermal conversion efficiency.^6−8^ Recently, photothermal membrane distillation has achieved a relatively higher vapor-to-distillate conversion efficiency and resolved the issue of salt accumulation, but the cost is too high owing to its complicated design of the photothermal active layer, poor durability, and external energy required.^9^ Therefore, it is urgent to develop a cost-efficient and sustainable strategy of freshwater production to resolve the problem of clean water shortage.

Solar-driven interface evaporation, based on localizing the heat from solar energy on the surface of photothermal materials, is deemed as a promising and renewable technology of clean water production.^10−12^ The photothermal materials have been widely investigated in recent years, which includes plasmonic particles, semiconductors, carbonaceous, and polymeric materials.^13−17^ According to investigations, carbon nanotubes (CNTs) are proposed as the ideal photothermal material owing to its outstanding light absorption of 0.98–0.99 in the broadband spectrum and large solar energy-to-thermal conversion efficiency.^18−25^ The CNT arrays prepared by chemical vapor deposition method exhibited extremely large solar thermal conversion efficiency of 90%,^26^ while the high cost limits its large-scale application in the field of water purification. Partial replacement of CNT and keeping the excellent performance would be a good strategy. CNT/PAN fabric, CNT/GO origami, and corn stalk/multiwalled carbon nanotubes (MWCNTs)/TiO_2_ based evaporators receive a high evaporation efficiency of 80% under solar irradiation of 1 kW m^–2^ (1 sun).^27−29^ It is worth noting that these outstanding evaporative performances mainly depend on the advanced structure design of the evaporator based on supporter, and CNTs just work as common solar photothermal powders in the hybrid structures and ignore its unique one-dimensional structure, resulting in lowering its advantages. Therefore, a good construct of CNT evaporator with a smart water pathway and excellent thermal management is highly desired. Li’s group reported hollow CNT-aerogel synthesized by carbonization of conjugated microporous polymer nanotubes via complex steps, which made full use of the advantages of CNTs and obtained a high evaporation efficiency 86.8% under 1 sun illumination.^30^ Hence, it is a challenge to exploit a cost-efficient synthesis of well-designed CNT evaporator with tunable water transport path and desired porosity to accelerate the water evaporation.

Here, we report a vertically arranged CNT (V-CNT) aerogel with a tree branch structure for efficient solar steam generation by a facile ice templating method (Figure S1).^31,32^ Ice templating is used to introduce a unique pore morphology into porous scaffolds through introduction of unidirectional ice into a fluid material. It is worth noting that the growth of ice crystals is perpendicular under temperature gradient from the bottom to top when the cooling process begins, and the particles gather along the vertical ice crystals. After freeze-drying, CNT aerogels with vertically arranged porous clusters and smaller bifurcating branches on the top layer are obtained, which provide numerous vertical channels to facilitate the transportation of water molecules and cut off the heat transfer to the bottom water. With the advantages of outstanding photothermal conversion of CNTs, the three-dimensional (3D) V-CNT evaporator (Figure S1) exhibits an impressive evaporation rate of 3.26 kg m^–2^ h^–1^ under 1 sun illumination, which is promising for practical solar evaporation and large-scale water purification.

## 2 Experimental Section

### 2.1 Preparation of V-CNTs

#### 2.1.1 Materials

MWCNTs (95%, length < 10 μm and diameter ∼ 50 nm) were obtained from Nanjing Xianfeng Nanomaterials Technology Co., LTD. Glutaric dialdehyde (C_5_H_8_O_2_, 25%) was purchased from Shanghai Macklin Biochemical Technology Co., LTD. Polyvinyl alcohol (PVA, *D*_p_ = 1750 ± 50, AR), hydrochloric acid (HCl, AR), sulfuric acid (H_2_SO_4_, AR), and nitric acid (HNO_3_, AR) were produced by Sinopharm Chemical Reagent Co., Ltd.

#### 2.1.2 Modification of CNTs

As shown in Figure S2, 1.0 g of MWCNTs were dispersed in 25 mL of sulfuric acid by ultrasonic vibration for 1 h, and then 25 mL of nitric acid was dropped in the mixture under vigorous stirring and reflux condensation at 85 °C for 100 min. The solution was separated by a centrifuge (TG16) with 10000 r/min and washed with deionized water many times until the pH value of the solution was 7. Finally, the black precipitate was dried under vacuum at 60 °C for 12 h to obtained the modified CNTs.

#### 2.1.3 Preparation of V-CNT Aerogel

**Figure 1:**
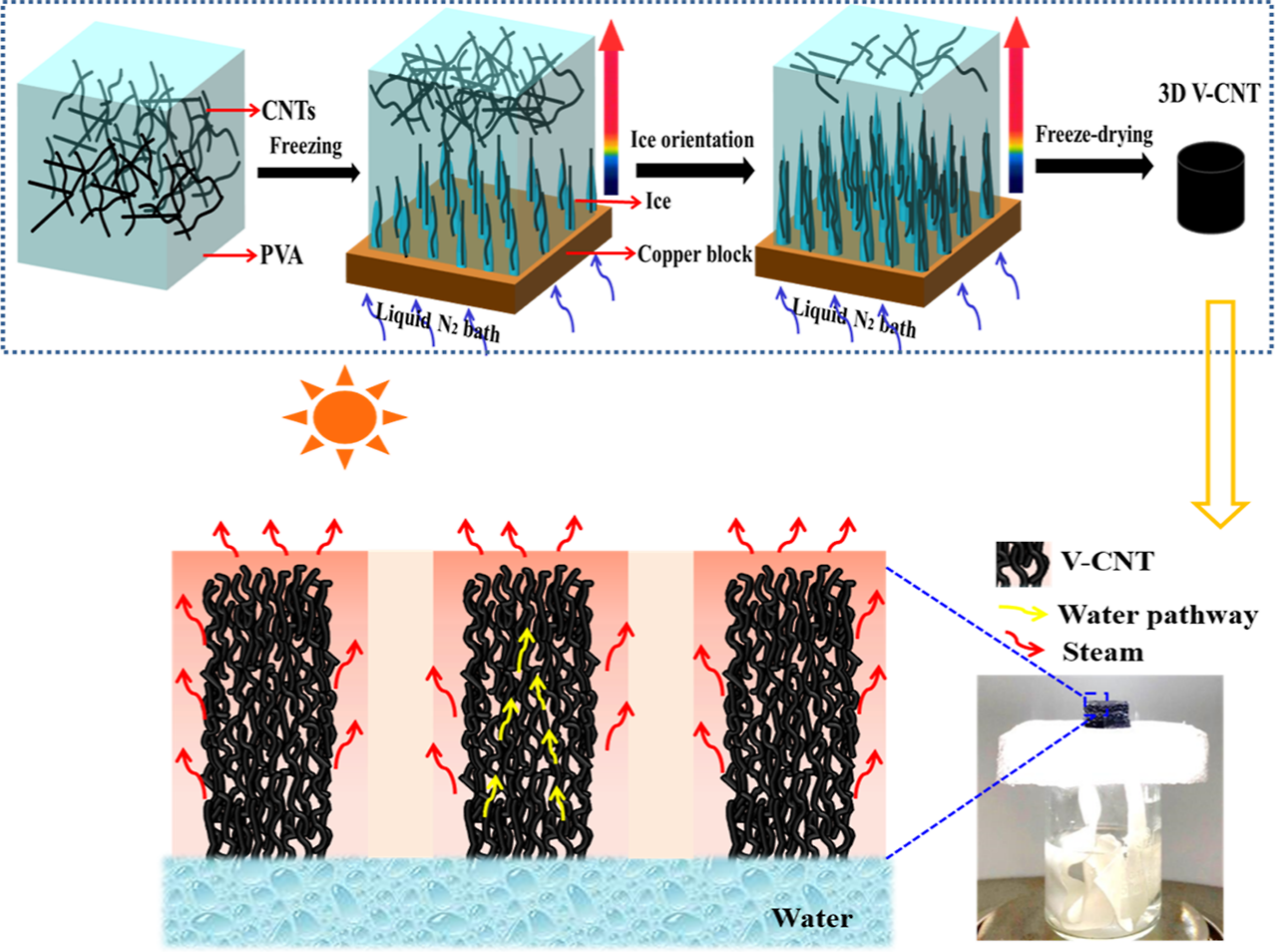
Schematic preparation of V-CNT via ice templating for efficient solar evaporation.

First, 2 mL of pre-cross-linking PVA solution (2.5 wt %, Figure S3) was dropped into 0.15 g of modified CNTs, followed by ultrasound for 1 h and stirring for 30 min. Second, the viscous slurry was transferred to a polytetrafluoroethylene container (25 mm in diameter) and then loaded on a copper block, which was immersed in liquid nitrogen for ice crystals growing vertically in 15 min. The arranged, ordered CNT skeletons were constructed by controlling the growth direction of ice crystals in the vertical direction (Figure 1). After freeze-drying, the V-CNT aerogel was obtained, followed by annealing at 100 °C for 30 min to reinforce its mechanical property via aging the cross-linking network of PVA. The obtained vertical arranged CNT aerogel was defined as V-CNT.

### 2.2 Characterization

The surface morphology of the V-CNT was examined by field emission scanning electron microscopy (Regulus 8200, Hitachi). The crystal structure was characterized by an X’Pert Pro MPD X-ray diffractometer (Panalytical, Holland). Optical absorption rate (A %) was recorded by a UV–vis–NIR spectrometer equipped with an integrating sphere (Lambda 750s, PerkinElmer). A contact angle detector (DSA 30, Krüss) was used to characterize the surface-wetting property of the sample. The ion concentration of water was tracked by inductively coupled plasma atomic emission spectrometry (ICP-OES, ThermoFisher Scientific iCAP 7400).

### 2.3 Solar Steam Generation Experiment

The solar steam generation experiment was carried out in the lab with room temperature of 25 °C and environmental humidity of ∼50%. The V-CNT aerogel (ϕ = 17 mm) was put on the groove of an EPE foam with a “U-shape” airlaid paper contacting the bulk water, which prevents the heat transfer from the top photothermal layer to the bulk water. The irradiation was carried out by a simulated solar (xenon light, CEL-S500, Education Au-light Co Beijing, China). The power of simulated solar was adjusted by a calibrated power meter (1918-R, Newport). The variation of mass was recorded on real time by a computer combined with an electronic balance (AR224CN, OHAUS, America). The surface temperature of the photothermal layer was measured by an infrared radiation camera (T1040, Flir) at various illumination times.

## 3 Results and Discussion

**Figure 2:**
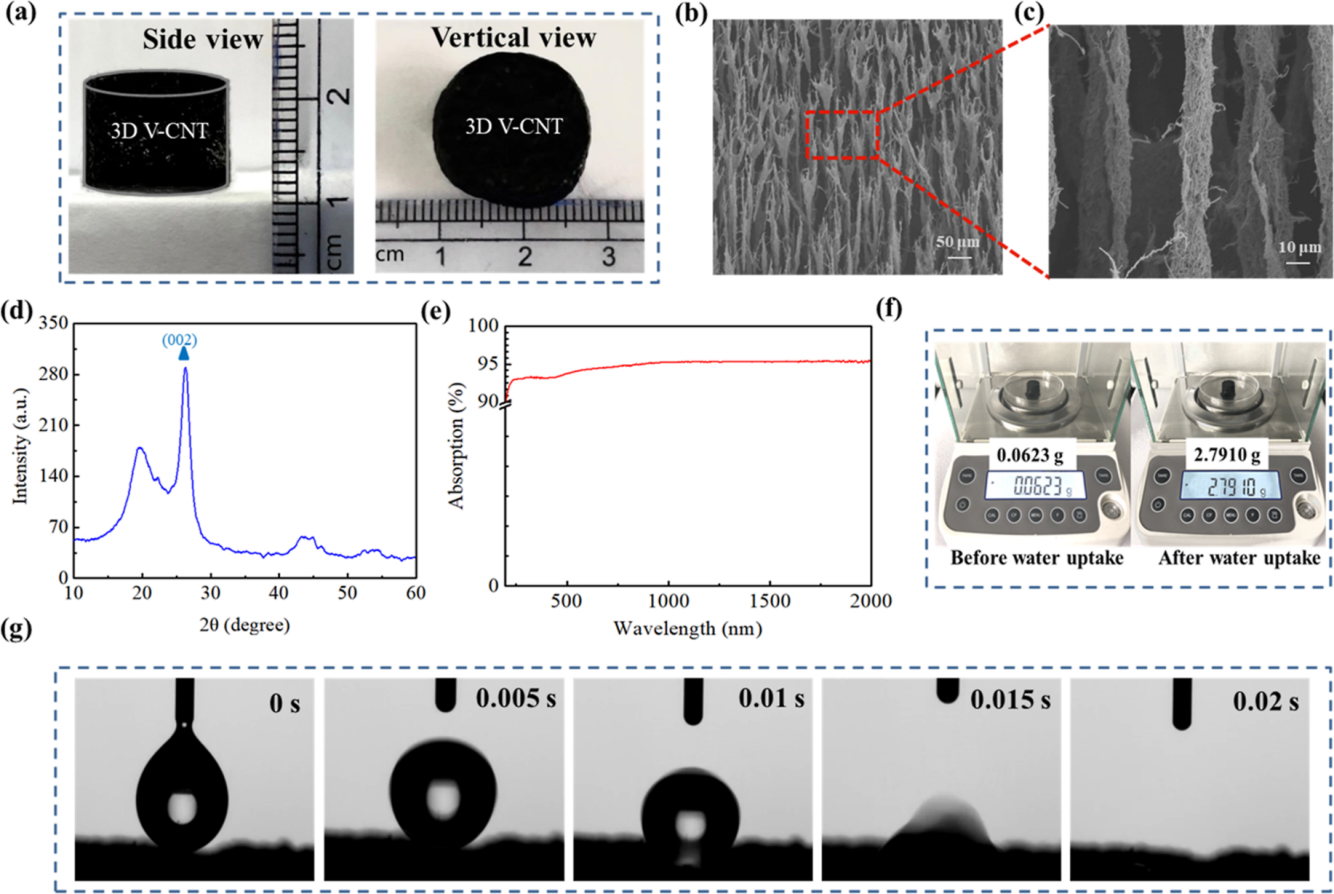
(a) Photographs of 3D V-CNT (ϕ = 17 mm, *h* = 12 mm). (b,c) SEM images of the 3D V-CNT. (d) XRD pattern of the V-CNT. (e) Absorption of dry V-CNT at the wavelength of 200–2000 nm. (f) Digital photographs illustrating the mass change of 3D V-CNT before and after water uptake. (g) Dynamic contact angles of the V-CNT at different times.

The V-CNT aerogel was prepared by ice templating as shown in Figure 1. As the cooled mixture of CNTs and PVA freezes, the CNTs gather around the vertical ice crystals owing to the temperature gradient.^33^ Then, the ice is removed by sublimation. The final microarchitecture consists of a pore morphology with vertically arranged clusters. The obtained vertically arranged CNT aerogel with a diameter of 17 mm and height of 12 mm is shown in Figure 2a.

The microscopic structures of V-CNT are observed by SEM images (Figure 2b,c). A large number of CNTs vertically arrange to form porous cylinders with a diameter of ∼10 μm and develop to bifurcate smaller branches with few CNTs at the top. The unique structures of V-CNT are similar to that of a tree with numerous channels for water transportation and the top leaves for fast evaporation, indicating that V-CNT provides an ideal microstructure for efficient solar evaporation.

The XRD pattern of V-CNT is shown in Figure 2d, and the typical peak at 25.3° corresponds to the (002) plane of carbon.^34^ Excellent absorption is an important factor of solar evaporation. As shown in Figure 2e, the absorption of dry V-CNT is about ∼95% at the wavelength of 200–2000 nm. The lost absorption can be attributed to the extremely low reflectance (∼4.8%) and transmittance (∼0) (Figure S4).

Water transportation is the guarantee of continuous solar steam generation. As shown in Figure 2f, the V-CNT is able to hold ∼45 times its own weight of water, indicating that the V-CNT has outstanding water-holding capacity. This value is much higher than those of reported photothermal evaporators such as 3D carbon aerogel, photothermal reservoir, graphene, and rice-straw-fiber-based aerogels.^35−37^ The hydrophilicity of the V-CNT is measured by contact angle (Figure 2g). It can be observed that a droplet on the surface of V-CNT is absorbed in only 0.02 s, indicating that the V-CNT aerogel has excellent hydrophilicity. These results demonstrate that the V-CNT aerogel-based evaporator not only captures broadband sunlight but also provides continuous water transportation for evaporation.

**Figure 3:**
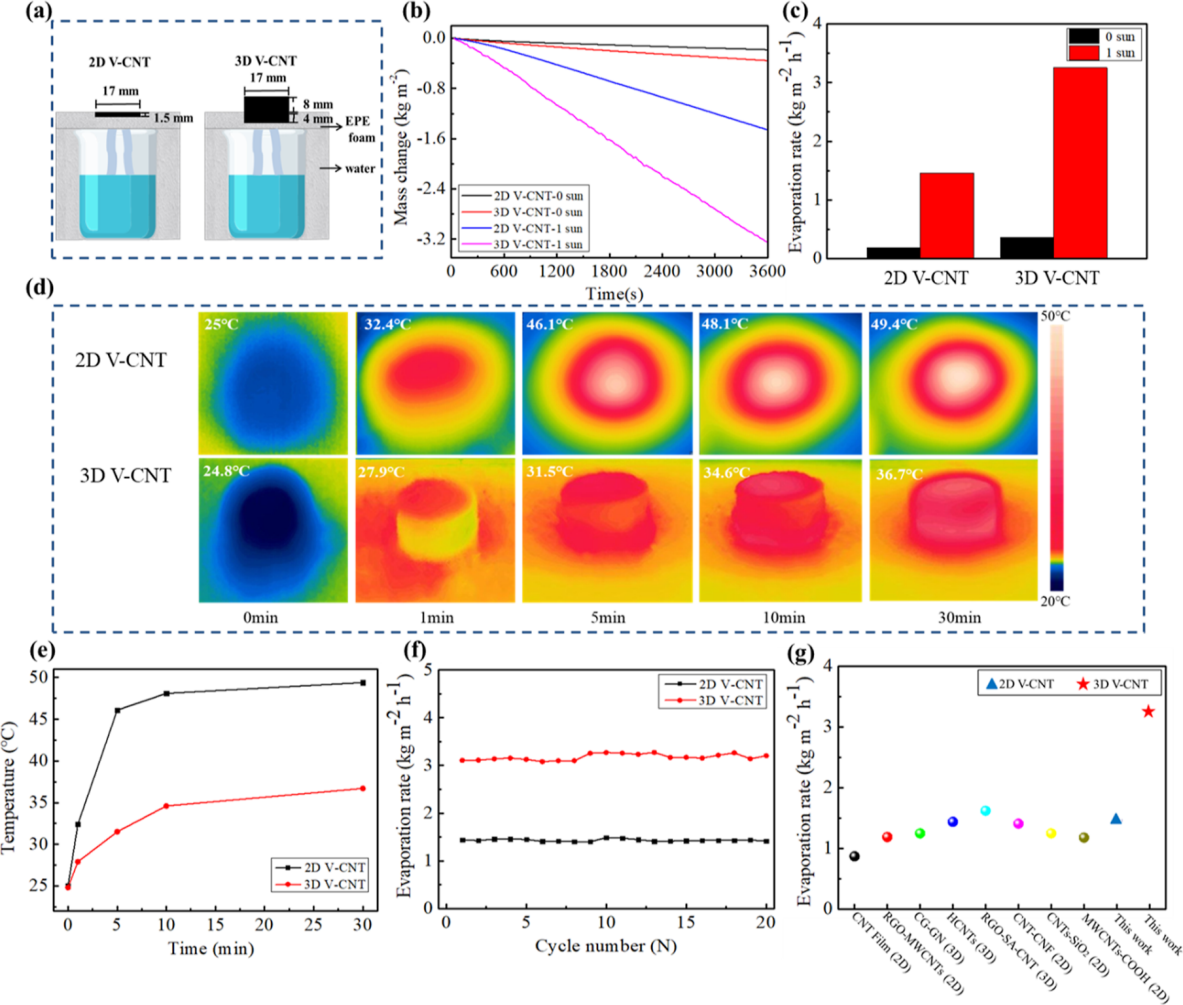
(a) Schematic illustration of evaporators: 2D V-CNT (ϕ = 17 mm, height = 1.5 mm) and 3D V-CNT (ϕ = 17 mm, height = 8 mm). Mass changes (b) and evaporation rates (c) of 2D and 3D V-CNT under 1 sun illumination and natural evaporation (0 sun), respectively. (d) Infrared images of 2D and 3D V-CNT during evaporation at various times. (e) Time-dependent change curves of the 2D and 3D V-CNT evaporators. (f) Cycling evaporation performance of V-CNT under 1 sun illumination with an interval of 30 min. (g) Comparison of evaporation rates between this work and reported CNT-based evaporators.

The evaporation experiments of V-CNT aerogels were carried out under 1 sun illumination at 25 °C with a relative humidity of 40–50% (Figure 3). The V-CNT-based evaporators are designed in 2D and 3D structures (Figure 3a). When the solar irradiation begins, the weight of water decreases steadily (Figure 3b). The mass flux (evaporation rate) of water can be calculated by the following equations
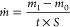
1where *m*_1_ and *m*_0_ are the initial and final mass of water, respectively, *t* is the illumination time, and *S* refers to the evaporation area. It can be observed that the evaporation rate of 3D V-CNT is 3.26 kg m^–2^ h^–1^ under 1 sun illumination, which is about 2.2 times that of 2D V-CNT (1.43 kg m^–2^ h^–1^) (Figure 3c). It can be ascribed to that the super-hydrophilicity and more dendritic structures of 3D V-CNT contribute to fast water transportation and larger evaporative area (Figure 1). The IR images of the V-CNT-based evaporator during the solar steam generation process at different times are shown in Figure 3d. Obviously, the temperature of 2D V-CNT increases sharply to 32.4 °C in 1 min and then rises to 49.4 °C after 30 min of illumination (Figure 3e). These values are much higher than those of reported 2D evaporator,^27,30^ implying that the V-CNT can transfer the absorbed sunlight to heat much more effectively than those traditional photothermal materials because the CNTs with a large aspect ratio have extremely high thermal conductivity in the longitudinal direction. In contrast to 2D V-CNT, the surface temperature of 3D V-CNT increases much slowly and maintains at 36.7 °C after 30 min of illumination, which can be attributed to the fact that the heat generated from the absorbed sunlight of the top surface transfers to the body and lateral surface of 3D V-CNT to achieve a larger evaporation area.

The stability of the V-CNT-based evaporator is important for long-term operation of water purification. Cycling performance of evaporation rates is taken 20 times under 1 sun illumination (Figure 3f). The evaporation rates of 2D and 3D V-CNT maintain at about 1.43 and 3.18 kg m^–2^ h^–1^, respectively, indicating that the V-CNT-based evaporators exhibit excellent stability and are promising for long-term solar evaporation. By comparing with the reported CNT-based evaporators, the evaporation rates of V-CNTs are much larger than those of reported CNT-based evaporators in 2D and 3D structures such as CNT-film, MWCNTs-COOH, RGO-MWCNTs, CG-GN, and HCNTs (Figure 3g).^27,30,38−43^ The evaporation mechanism of the efficient V-CNT evaporator is shown in Figure S5. The outstanding evaporation rates of V-CNT evaporators can be ascribed to the following two reasons. First, the super-hydrophilicity endows the water to transport upwardly to the evaporative layer along the V-CNT cluster, and the tree-like branch on the top surface (Figure 2) enlarges the evaporative area to facilitate more steam escape. Second, the width of vertical spaces between the CNTs clusters is 10∼20 μm, which fills with the air that not only cuts off the liquid thermal conduction but also helps the steam escape from the surface of CNT clusters (Figure 1) according to the above results of Figure 2. The unique structure of the V-CNT not only captures full spectrum solar energy but also lowers heat loss and provides enough water supply for efficient evaporation.

**Figure 4:**
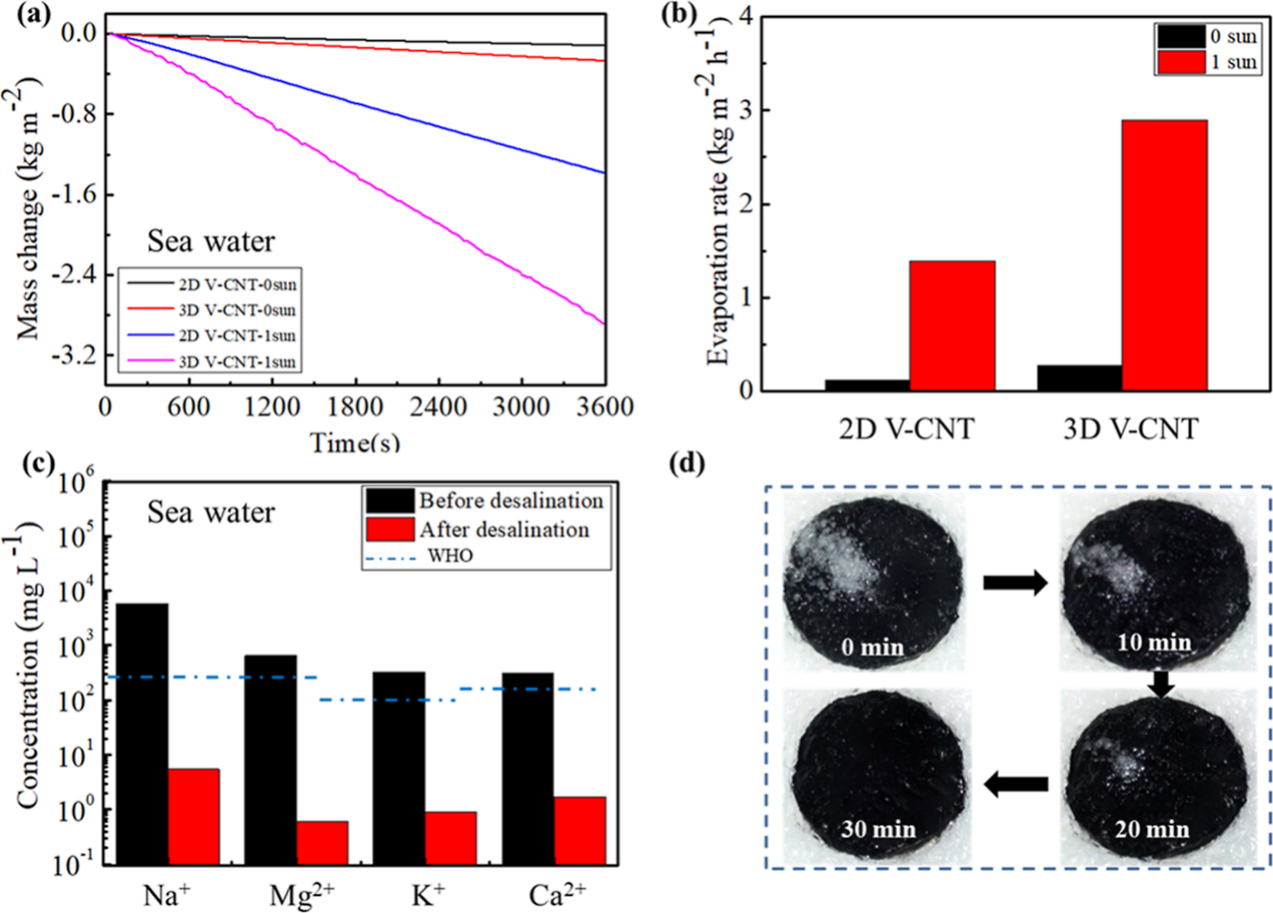
Seawater desalination of V-CNT-based evaporators. Mass changes (a) and corresponding evaporation rates (b) of 2D and 3D V-CNTs under 1 sun irradiation and 0 sun, respectively. (c) Comparing ion concentrations of actual seawater (Donghai, China) before and after evaporation; blue dotted lines are the salinity standards of WHO for drinkable water. (d) Digital images of the surface salt rejection from the 2D V-CNT during the seawater desalination at different times.

To evaluate the practical application of the V-CNT in seawater desalination, the solar steam generation of actual seawater (Donghai, China) was carried out under 1 sun irradiation at 25 °C with a humidity of 40–50%. Figure 4a shows that the mass change of seawater declines linearly, and the evaporation rates of 2D and 3D V-CNT are 1.39 and 2.90 kg m^–2^ h^–1^ (Figure 4b), respectively. The ion concentrations of the condensed vapor during the solar evaporation are characterized by ICP-OES (Figure 4c). Obviously, the concentrations of four primary ions (Na^+^, Mg^2+^, K^+^, and Ca^2+^) are significantly reduced by 2 to 3 orders and meet the standard of World Health Organization (WHO) for drinking water.^44^

**Figure 5:**
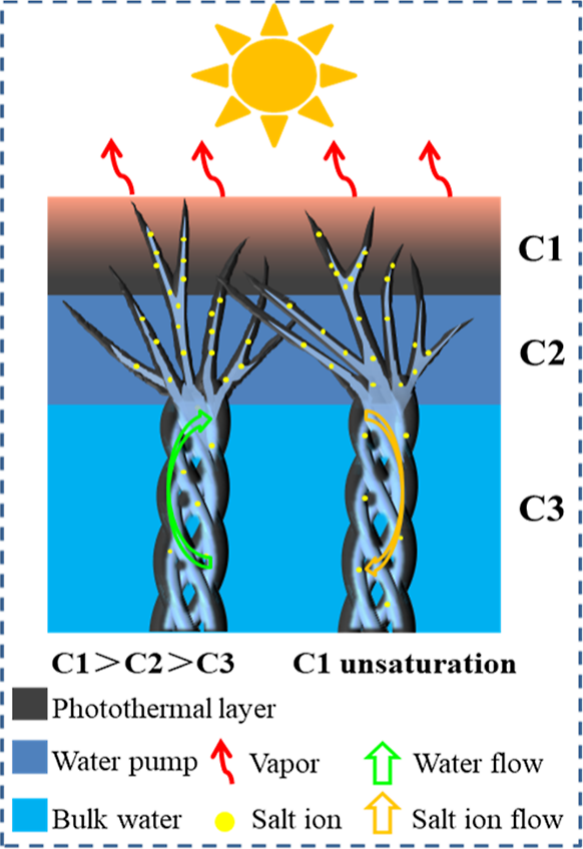
Mechanism of salt resistance in the V-CNT evaporator.

In order to reveal the mechanism of salt resistance, 0.06 g of NaCl crystals are put on the surface of the moist 3D V-CNT under 1 sun illumination (Figure 4d). Obviously, the NaCl crystals dissolve within 30 min, and the surface keeps moist, indicating that the V-CNT-based evaporator has an outstanding stability for long-term operation of seawater desalination. The reason may be ascribed to three reasons as follows. First, the super-hydrophilicity and outstanding water-holding capability of the V-CNT aerogel (Figure 2) provide sufficient water supply on the top layer to dissolve the solid salt crystals rapidly, leading to a high salt concentration (C1). Second, the gradient salt concentration (C1 > C2 > C3, Figure 5) was formed between the top and bottom of the evaporator,^45^ resulting in severe diffusion and convection in the evaporator. The hydrophilic wettability enables that the water wicking rate is much faster than the evaporation rate,^46^ leading to maintaining unsaturation of the top evaporative layer. Finally, the vertical arranged channels with open large holes (∼20 μm, Figure 2c) in the V-CNT aerogels also contribute to the transportation of salt ions back to the bulk water and keep the water pathway unblocked, which is similar to that of reported wood by Hu et al.^47,48^ Therefore, the V-CNT aerogels exhibit an excellent salt-resistant performance and is promising for application in practical seawater desalination.

## Conclusions

In summary, a V-CNT aerogel with a tree-like structure was prepared by a facile ice templating method for efficient solar steam evaporation. The V-CNT aerogel with vertical CNT clusters not only exhibits excellent hydrophilicity and water capacity but also decreases heat loss and keeps the water pathway open for delivering salt ions back to bulk water. The evaporation rate of 3D V-CNT is 3.26 kg m^–2^ h^–1^, which is about 2.2 times that of 2D V-CNT (1.46 kg m^–2^ h^–1^) under 1 sun irradiation. The unique structure of V-CNT exhibits an outstanding salt resistance in the long-time seawater evaporation. The ion concentrations of the purification water of seawater meet the WHO standard for drinking water. This work provides a promising photothermal material for efficient purification of seawater and sewage.

## Supporting Information Available

Photographs of V-CNT aerogel on the flowers; schematic preparation of modified MWCNTs; preparation of PVA solution; reflectance and transmittance of the dry V-CNT aerogel; solar evaporation mechanism of the solar evaporator; photographs of the 2D V-CNT- and 3D V-CNT-based evaporators; and infrared images of the 3D V-CNT during evaporation at different times (PDF)
