# Correction to “Facile Synthesis of Vertically Arranged CNTs for Efficient Solar-Driven Interfacial Water Evaporation”

**DOI:** 10.1021/acsomega.3c08339

**Published:** 2023-11-08

**Authors:** Lifen Su, Xiaoyu Liu, Xu Li, Bin Yang, Bin Wu, Ru Xia, Jiasheng Qian, Jianhua Zhou, Lei Miao

In the Acknowledgment, the right Funding number of National Natural Science Foundation of China should be No. 521032**95**.

