# Supplementary material for: Facile Synthesis of Vertically Arranged CNTs for Efficient Solar-Driven Interfacial Water Evaporation
Source: ACS Omega. 2022 Dec 12;7(50):47349–56. doi: 10.1021/acsomega.2c06706 (PMC9774377; doi:10.1021/acsomega.2c06706)
Supplement: Supplementary file 1 — ao2c06706_si_001.pdf [file ao2c06706_si_001.pdf]

Supporting information

Facile Synthesis of Vertically Arranged CNTs for Efficient Solar-driven Interfacial Water Evaporation

Lifen Su [a, b, §], Xiaoyu Liu [a, §], Xu Li [a], Bin Yang [a, *], Bin Wu [a, *], Ru Xia [a], Jiasheng Qian [a], Jianhua Zhou [c], Lei Miao [c, *]

[a] Anhui Province Key Laboratory of Environment-friendly Polymer Materials, School of Chemistry and Chemical Engineering, Anhui University, Hefei 230601, China

[b] School of Materials Science and Engineering, Anhui University, Hefei 230601, China

[c] Guangxi Key Laboratory of Information Materials, Engineering Research Center of Electronic Information Materials and Devices, Ministry of Education, Guilin University of Electronic Technology, Guilin 541004, China

E-mail: miaolei@guet.edu.cn (Lei Miao), beanyoung@163.com (Bin Yang), 20164@ ahu.edu.cn (Bin Wu)

§Lifen Su and Xiaoyu Liu contributed equally to this paper

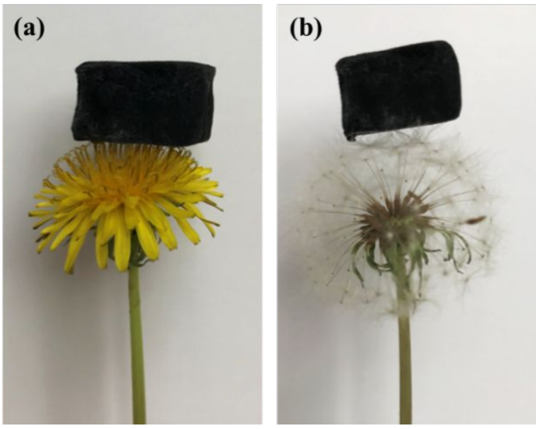

Figure S1. Photographs of V-CNT aerogel on the flowers: (a) dandelion corolla, (b) dandelion fruit.

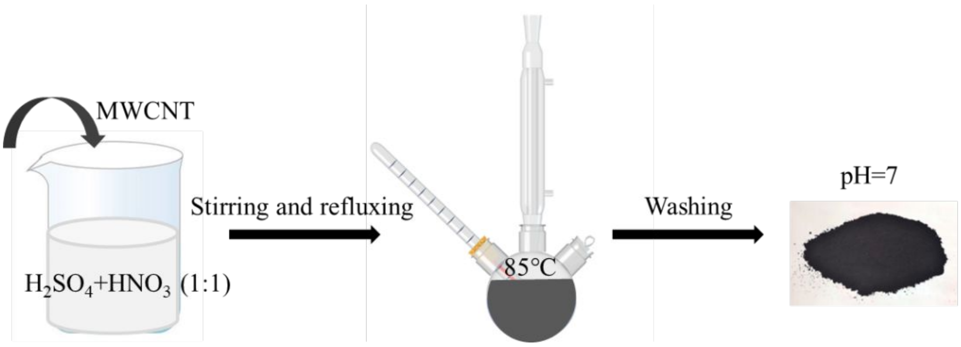

Figure S2. Schematic preparation of modified MWCNT

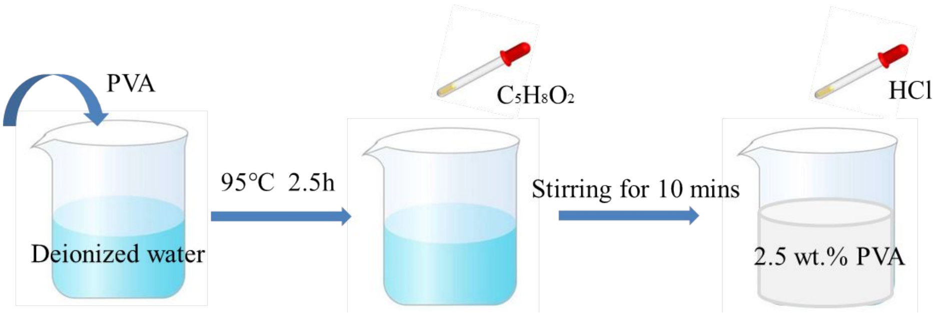

Figure S3. Preparation of PVA solution (2.5 wt.%)

The preparation of 2.5 wt.% PVA solution is shown in Figure S3. First, 1g PVA (D_p =1750 ± 50, Sinopharm Chemical Reagent Co., Ltd) was added into 40 mL deionized water and kept at 95°C for 2.5 hours. Second, 1mL glutaric dialdehyde ($C_5H_8O_2$, 1wt.%) was added into the transparent PVA solution under vigorous stirring at 25 °C. Finally, the dilute hydrochloric acid (HCl, 1mol L^{-1}) was added into the mixture under until pH=5, and further cross-linking reaction was carried out at 80 °C for 30 minutes. The pre-crosslinking PVA solution was obtained.

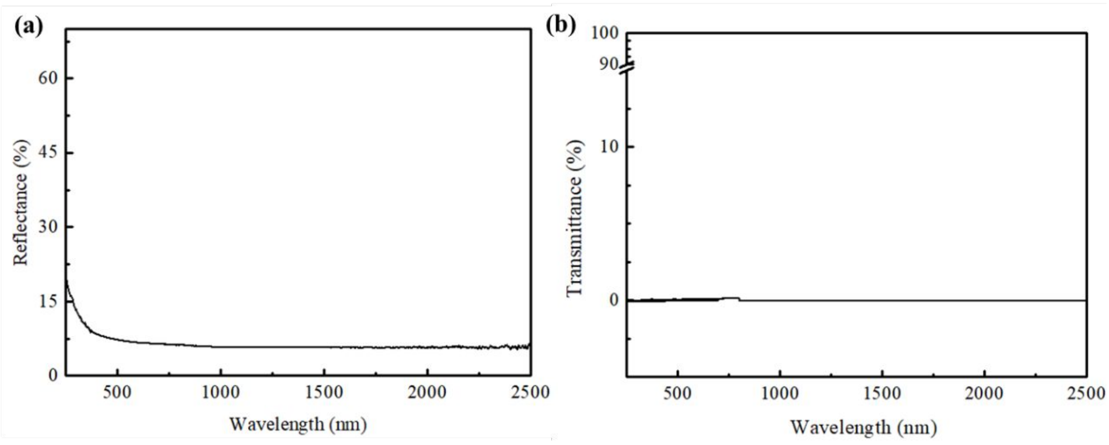

Figure S4. The reflectance (a) and transmittance (b) of the dry V-CNT aerogel

The reflectance (R%) and transmittance (T%) of the dry V-CNT aerogel in the wavelength range of 250-2500 nm are shown in Figure S4. It can be seen that the reflectance of V-CNT is about 5%～7.5% and the transmittance is close to zero. The absorption (A%) was calculated according to the equation of A%=100%−R%−T%.

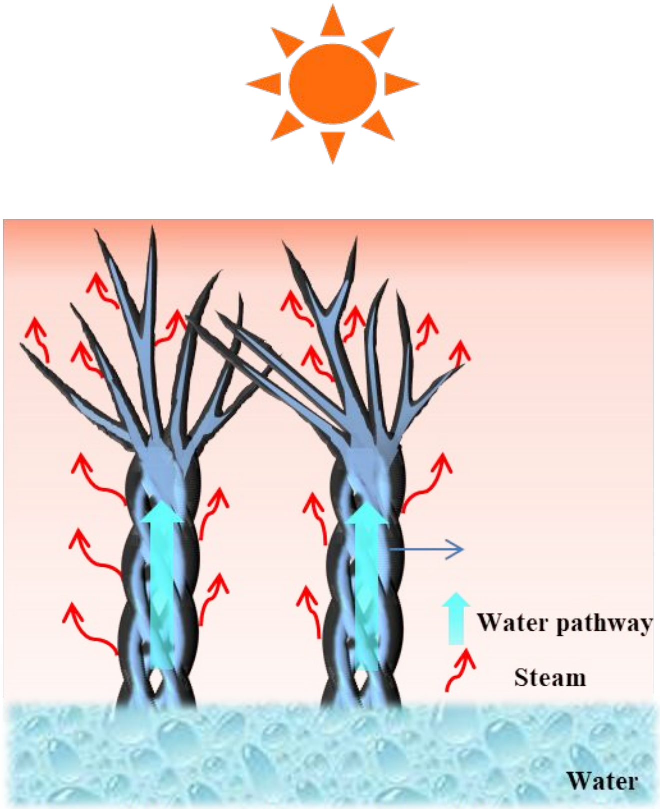

Figure S5. Solar evaporation mechanism of the solar evaporator.

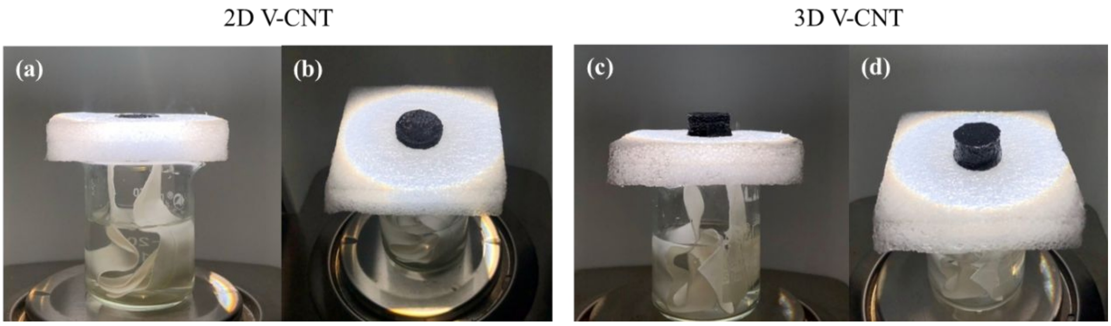

Figure S6. Side (a) and top surface (b) of 2D-CNT (ϕ=17 mm, h=1.5 mm) based evaporator; Side (c) and top surface (d) of 3D-CNT (ϕ=17 mm, h=8 mm) based evaporator.

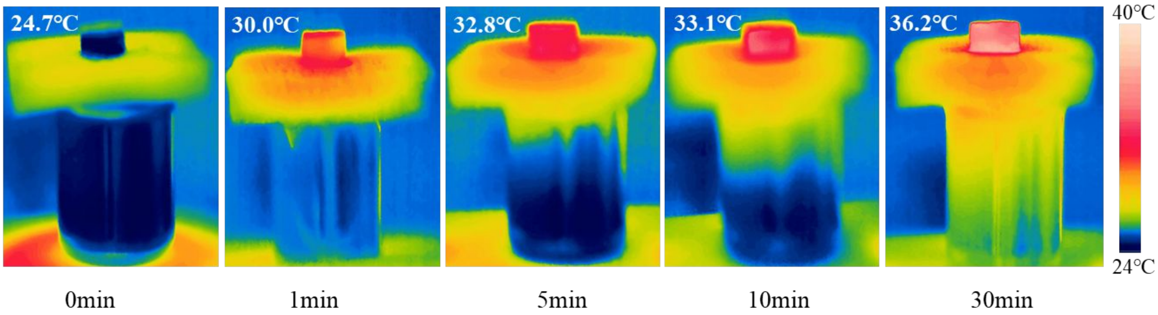

Figure S7. Infrared images of the 3D V-CNT during evaporation at different times.

As shown in Figure S7, the top temperature of 3D V-CNT increases sharply to 30.0 °C after illumination for 1 min, then keeps at 36.2 °C after 30 minutes. However, the bottom temperature of 3D V-CNT closes to that of the top, implying that the 3D V-CNT transfer some of absorbed solar energy from top to the body and achieve a larger evaporation rate.
